# Effect of Recruitment Methods on Response Rate in a Web-Based Study for Primary Care Physicians: Factorial Randomized Controlled Trial

**DOI:** 10.2196/jmir.8561

**Published:** 2018-02-08

**Authors:** Ryuhei So, Kiyomi Shinohara, Takuya Aoki, Yasushi Tsujimoto, Aya M Suganuma, Toshi A Furukawa

**Affiliations:** ^1^ Department of Health Promotion and Human Behavior Graduate School of Medicine/School of Public Health Kyoto University Kyoto Japan; ^2^ Okayama Psychiatric Medical Center Okayama Japan; ^3^ Department of Healthcare Epidemiology Graduate School of Medicine/School of Public Health Kyoto University Kyoto Japan

**Keywords:** Web-based surveys, electronic mail, incentives, surveys and questionnaires, survey methods, questionnaire design, data collection, physicians, general practitioners

## Abstract

**Background:**

Low participation rates are one of the most serious disadvantages of Web-based studies. It is necessary to develop effective strategies to improve participation rates to obtain sufficient data.

**Objective:**

The objective of this trial was to investigate the effect of emphasizing the incentive in the subject line of the invitation email and the day of the week of sending the invitation email on the participation rate in a Web-based trial.

**Methods:**

We conducted a 2×2 factorial design randomized controlled trial. We contacted 2000 primary care physicians from members of the Japan Primary Care Association in January 2017 and randomly allocated them to 1 of 4 combinations of 2 subject lines (presence or absence of an emphasis on a lottery for an Amazon gift card worth 3000 yen or approximately US $30) and 2 delivery days (sending the invitation email on Tuesday or Friday). The primary outcome was the response rate defined as the number of participants answering the first page of the questionnaire divided by the number of invitation emails delivered. All outcomes were collected between January 17, 2017, and February 8, 2017.

**Results:**

We analyzed data from 1943 out of 2000 participants after excluding those whose email addresses were invalid. The overall response rate was 6.3% (123/1943). There was no significant difference in the response rates between the 2 groups regarding incentive in the subject line: the risk ratio was 1.12 (95% CI 0.80 to 1.58) and the risk difference was 0.7% (95% CI –1.5% to 2.9%). Similarly, there was no significant difference in the response rates between the 2 groups regarding sending the email on Tuesday or Friday: the risk ratio was 0.98 (95% CI 0.70 to 1.38) and the risk difference was –0.1% (95% CI –2.3% to 2.1%).

**Conclusions:**

Neither emphasizing the incentive in the subject line of the invitation email nor varying the day of the week the invitation email was sent led to a meaningful increase in response rates in a Web-based trial with primary care physicians.

**Trial Registration:**

University Hospital Medical Information Network Clinical Trials Registry UMIN000025317; https://upload.umin.ac.jp/cgi-open-bin/ctr_e/ctr_view.cgi?recptno=R000029121 (Archived by WebCite at http://www.webcitation. org/6wOo1jl9t)

## Introduction

One of the most serious drawbacks of Web-based studies is their considerably smaller participation rates compared to paper-based studies [1,2], even though Web-based studies are widely used to explore clinicians’ knowledge, perspectives, and clinical practice [3-5]. In a meta-analysis examining participation rates of studies targeting clinicians, the mean participation rate had decreased over the years [1]. Furthermore, participation rates of 20% or less are not uncommon in Web-based studies especially for physicians [1]. These low participation rates may impair the precision of estimates due to reduced sample size and call into question representativeness of the subject group, leading to selection bias. Thus, researchers planning Web-based studies need to develop effective strategies to improve participation rates to obtain sufficient data.

Previous studies have investigated factors related to participation rates in Web-based studies. Incentives, contact timing, content of subject line and message of invitation emails, length of questionnaire, survey webpage design, and individualization among others were proposed as factors that may improve participation rates of Web-based studies [6-8]. Among these, incentives, contact timing, and content of invitation emails were considered the most useful because they require no special system. In studies targeting clinicians, incentives were considered to increase participation rates, and monetary incentives were reported to be more effective than nonmonetary incentives and no incentive [6,9-11]. However, whether emphasizing incentives in the subject line of an invitation email increases participation rates remains unclear. Two studies have reported that participation rates of Web-based studies have decreased by emphasizing incentives in the subject line among college students or general population adults [12,13]. Alternatively, Janke et al [14] reported that an invitation email emphasizing a monetary incentive in the subject line significantly improved response rate (10.4% vs 5.6%, *P*=.03) among college students. We do not yet know whether clinicians show similar patterns. To our knowledge, the effect of emphasizing incentives in the subject line of invitation emails for clinicians has not been studied.

As for contact timing, previous studies have reported conflicting findings regarding the effect of the day of the week of sending postal mail invitations on study participation rates among general practitioners [15]. One study found that the weekend was more effective than weekdays for postal mailing [16], but 2 others did not [17,18]. The effect of the day of sending invitations by email has not been examined to date, whereas this has been done with postal mail [15].

The objective of this trial was to investigate the effect of emphasizing the incentive in the subject line of the invitation email and the day of the week of sending the invitation email on the participation rate in a Web-based trial.

## Methods

### Design

We conducted a 2×2 factorial design randomized controlled trial (RCT) to test 2 recruitment strategies for the invitation email in a Web-based trial with participants who were members of the Japan Primary Care Association (JPCA).

### Procedures

This study was a substudy regarding the recruitment process embedded in another randomized controlled study (Do Overstated Conclusions Trick Our Readers? The DOCTOR study [UMIN000025317]), which investigated whether overstated conclusions in abstracts of clinical research papers can bias primary care doctors’ impressions of the results. The DOCTOR study was conducted between January 17, 2017, and February 8, 2017. In the DOCTOR study, participants were asked to complete a 2-page online questionnaire about evidence-based medicine. The questionnaire had 9 and 4 items, respectively, on the first and second pages. A completeness check was automatically conducted after the questionnaire was submitted and respondents were asked to complete the mandatory items if they were blank. The first researcher (RS) developed all the Web systems for the DOCTOR study. Details of the methods and results of the DOCTOR study are reported elsewhere [19].

To maximize the number of respondents in the DOCTOR study, we recruited the participants in 2 phases. In the first phase, we randomly sampled 2000 primary care physicians from 7040 potential participants and sent the invitation email to investigate the most effective subject line (emphasizing an incentive of a chance to win an Amazon gift card worth 3000 Japanese yen [US $30]; Amazon gift cards were to be provided to 20 respondents through a lottery) on different days of the week (Tuesday vs Friday). In the second phase, we invited remaining potential participants using the most effective method confirmed in the first phase. We hereby report the results from the first phase. The English version of the invitation email is included in the supplementary appendix reported elsewhere [19].

### Interventions

We tested the 2 interventions related to the invitation email. One intervention involved mentioning the chance to receive an Amazon gift card in the subject line (“[Get Amazon gift card] Survey on EBM for 5 minutes”) or not (“[Funded by the association] Survey on EBM for 5 minutes”). The other intervention involved sending the invitation email at 6:00 PM on either Tuesday, January 17, 2017, or Friday, January 20, 2017.

### Participants

The eligibility criteria for participation were as follows: being a member of the JPCA, which is the largest association of primary care doctors in Japan; having more than 2 years of clinical practice experience; and having a registered email address with the JPCA. Eligible participants of the DOCTOR study were 7040 primary care doctors out of the 10,851 members of the JPCA in January 2017 when the DOCTOR study was conducted.

To limit participants to members of the JPCA, we did not announce the DOCTOR study on websites or social networking services with open access. The recruitment process was conducted only through emails from the secretariat of the JPCA, which invited doctors to voluntarily participate in the study.

### Randomization and Blinding

We randomly assigned a sample of 2000 primary care physicians who were randomly selected from the eligible JPCA members to 4 groups (500 participants in each group): (1) those who received the invitation email with a subject line emphasizing the incentive on Tuesday, (2) those who received the invitation email with a subject line emphasizing the incentive on Friday, (3) those who received the invitation email with a subject line not emphasizing the incentive on Tuesday, and (4) those who received the invitation email with a subject line not emphasizing the incentive on Friday.

The invitation emails were sent by the secretariat of the JPCA according to the allocation table prepared by RS using a computer-generated random sequence. Potential participants were not informed that the invitation emails were sent in 4 different ways. Outcomes such as response, completion, and access to the DOCTOR study website were automatically recorded.

### Outcomes and Data Collection

The primary outcome was the response rate defined as the number of participants completing the first page divided by the number of invitation emails delivered. The formula was equivalent to Response Rate 2, defined by the American Association for Public Opinion Research [20], which was used in a previous study investigating strategies to improve response rates for a Web-based clinician study [21].

Secondary outcomes were the completion rate and access rate to the DOCTOR study website. The completion rate was defined as the number of respondents who completed all the pages on the DOCTOR study website divided by the number of invitation emails delivered. The access rate was the number of participants accessing the DOCTOR study website divided by the number of invitation emails delivered.

The data were collected between January 17, 2017, and February 8, 2017. The response rate and completion rate were recorded by a Web application developed by RS for the DOCTOR study. The access rate was recorded using Google Analytics (Google). The allocation of each participant was tracked using a URL link unique to each allocation embedded in the invitation email. We used an IP address and a cookie to avoid double-counting the same user if any of them accessed or responded more than once. No bug fixes, downtime, or content changes were necessary during the period of the DOCTOR study.

### Sample Size

We included 2000 of the potential participants of the DOCTOR study in this factorial RCT to apply the most effective invitation email strategy to the remaining potential participants. With a sample size of 2000, we calculated the power to be 0.81 at an alpha level of .05 to detect the significant absolute risk difference of 3% against an assumed control response rate of 4% based on results of previous surveys targeting JPCA members.

### Data Analysis

Statistical analyses were performed using R version 3.3.1 (The R Foundation). To compare the response rates, completion rates, and access rates between groups, we reported both risk ratios and risk differences with 95% confidence intervals using the chi-squared test. We also compared demographics of respondents between the intervention groups. We used the chi-squared test for dichotomous variables and Student *t* test for continuous variables. We set the threshold of statistical significance at *P* ≤.05. All analyses were conducted according to the intention-to-treat principle.

### Ethics and Informed Consent

The DOCTOR study was approved by the Institutional Review Board (IRB) of Kyoto University Graduate School of Medicine. We did not include the present factorial RCT in the DOCTOR study when applying for an ethics review by the IRB because the aims of the study were not directly related to health care. The Board of Directors of the JPCA approved this factorial RCT in the recruitment processes of the DOCTOR study. The participants of the DOCTOR study provided their consent on the website.

To avoid the risk of personal information leak, we did not obtain identifiable personal information from participants other than winners of the incentive lottery. In addition, identifiable personal information of the winners was stored separately from the research data.

### Trial Registration

The DOCTOR study was registered prospectively at University Hospital Medical Information Network Clinical Trials Registry [UMIN000025317].

## Results

### Participant Flow

**Figure 1:**
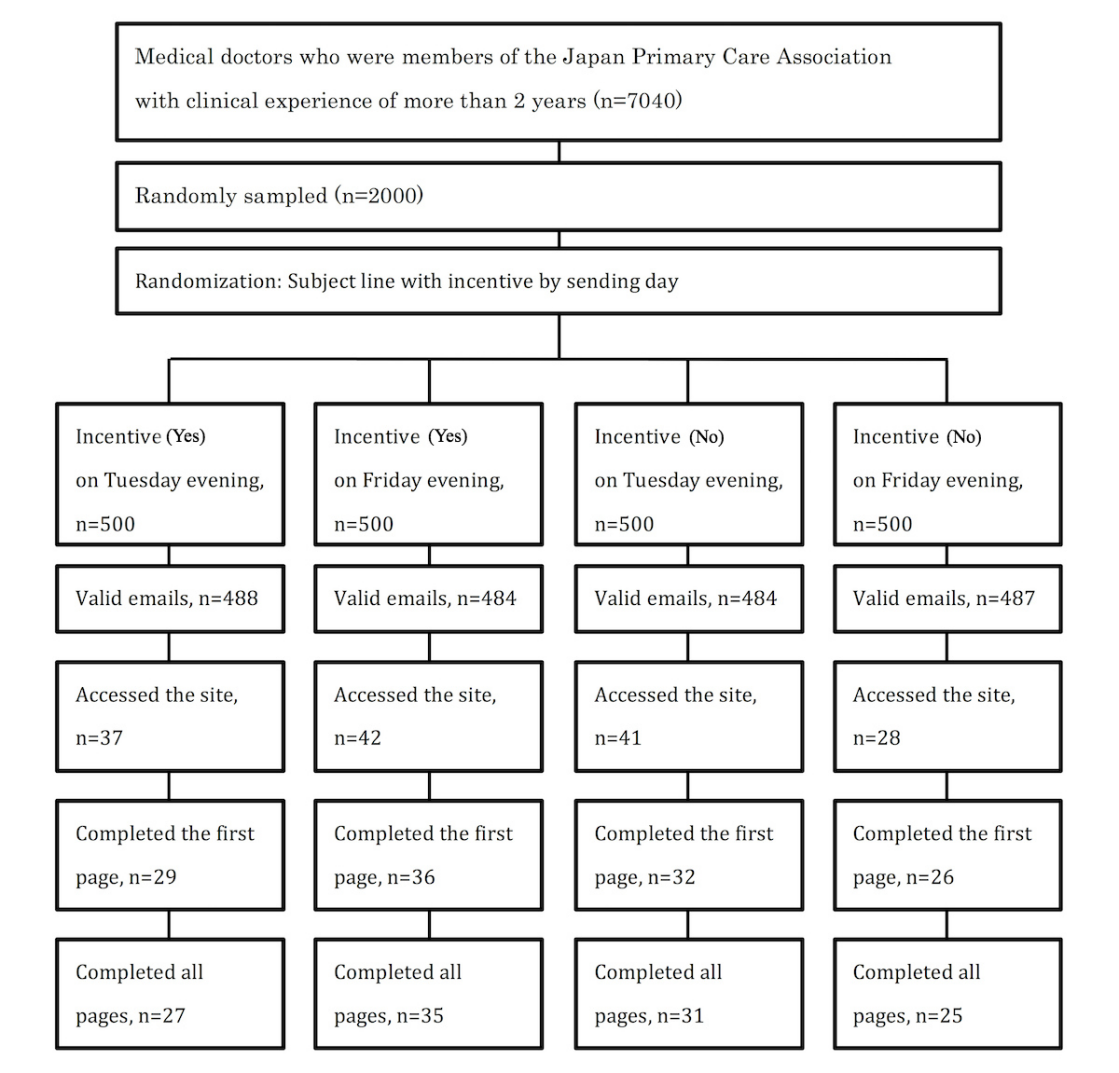
Flow diagram of participants.

Figure 1 shows the participant flow. Out of 2000 invitation emails, 57 were undeliverable; therefore, we analyzed data from the remaining 1943 participants for all outcomes. Of 1943 primary care doctors, 148 (7.61%) accessed the study site, 123 (6.33%) responded, and 118 (6.07%) completed the questionnaire.

### Characteristics of Respondents

Table 1 shows the characteristics of the 123 respondents. We did not find statistically significant differences in the characteristics among the 4 groups except for primary workplace at the nominal *P* value of .01 without adjusting for multiple comparisons.

### Access, Response, and Completion Rate on a Two-Level Factorial Basis

Access, response, and completion rates in each randomized group (Tuesday or Friday × incentive in the subject line or not) are reported with 95% confidence intervals in Table 2. We found no statistically significant difference in all the outcomes among the 4 groups. The interaction effects on all the outcomes between the 2 factors were not statistically significant. In the following sections, we therefore report the effect of the 2 interventions separately.

### Effect of Subject Line of the Invitation Email Emphasizing Amazon Gift Card Incentive

Table 3 summarizes the rates of access, response, and completion. The response rates, which were the primary outcome of this study, were 6.7% (65/972) in the intervention group and 6.0% (58/971) in the control group, respectively. There was no significant difference in the response rate between the 2 groups: the risk ratio was 1.12 (95% CI 0.80 to 1.58) and the risk difference was 0.7% (95% CI –1.5% to 2.9%). Similarly, the completion rate and access rate were almost the same in the 2 groups (see Table 3).

### Effect of the Day of the Week of Sending the Invitation Email

Table 4 shows all outcomes regarding the day of the week of sending the invitation email. The response rate was 6.3% (61/972) on Tuesday evening and 6.4% (62/971) on Friday evening.

No significant difference was demonstrated between the Tuesday group and Friday group: the risk ratio was 0.98 (95% CI 0.70 to 1.38) and the risk difference was –0.1% (95% CI –2.3% to 2.1%).

**Table 1:** Characteristics of respondents (n=123).

Characteristic		Tuesday		Friday			*P* value
		Incentive in the subject line	No incentive in the subject line		Incentive in the subject line	No incentive in the subject line	
Gender, male, n (%)		29 (86.2)	32 (93.8)		36 (91.7)	26 (92.3)	.76
Years of practice, mean (SD)		18.8 (9.8)	18.7 (9.8)		18.8 (9.9)	16.0 (8.7)	.63
**Workplace, n (%)**							.01
	University hospitals	3 (10.3)	6 (18.8)		5 (13.9)	5 (19.2)	
	Hospitals (public and private)	17 (58.6)	17 (53.1)		15 (41.7)	13 (50.0)	
	Clinics		3 (9.4)		16 (44.4)	8 (30.8)	
	Academic research institutes	0 (0.0)	4 (12.5)		0 (0.0)	0 (0.0)	
	Others	0 (0.0)	2 (6.2)		0 (0.0)	0 (0.0)	
**Degree/certification, n (%)^a^**							
	PhD	8 (27.6)	5 (15.6)		14 (38.9)	8 (30.8)	.20
	Primary care	21 (72.4)	23 (71.9)		20 (55.6)	13 (50.0)	.18
	Family medicine	4 (13.8)	5 (15.6)		7 (19.4)	10 (38.5)	.10
	Other certification	18 (62.1)	23 (71.9)		19 (52.8)	13 (50.0)	.29

^a^Duplicate responses allowed.

**Table 2:** Access, response, and completion rate in 4 randomized groups (Tuesday or Friday × incentive in the subject line or not).

Characteristic	Tuesday, n (%; 95% CI)		Friday, n (%; 95% CI)	
	Incentive in the subject line (n=488)	No incentive in the subject line (n=484)	Incentive in the subject line (n=484)	No incentive in the subject line (n=487)
Access	37 (7.6; 5.4 to 10.3)	41 (8.5; 6.1 to 11.3)	42 (8.7; 6.3 to 11.5)	28 (5.7; 3.9 to 8.2)
Response	29 (5.9; 4.0 to 8.4)	32 (6.6; 4.6 to 9.2)	36 (7.4; 5.3 to 10.1)	26 (5.3; 3.5 to 7.7)
Completion	27 (5.5; 3.7 to 7.9)	31 (6.4; 4.4 to 9.0)	35 (7.2; 5.1 to 9.9)	25 (5.1; 3.3 to 7.5)

**Table 3:** Effect of emphasizing the incentive in the subject line on study participation.

Characteristic	Incentive in the subject line, n (%; 95% CI)		Risk difference, % (95% CI)
	Yes (n=972)	No (n=971)	
Access	79 (8.1; 6.5 to 10.0)	69 (7.10; 5.6 to 8.9)	1.0 (–1.3 to 3.4)
Response	65 (6.7; 5.2 to 8.4)	58 (6.0; 4.6 to 7.7)	0.7 (–1.5 to 2.9)
Completion	62 (6.4; 4.9 to 8.1	56 (5.8; 4.4 to 7.4)	0.6 (–1.5 to 2.7)

**Table 4:** Effect of sending day of the invitation email on study participation.

Characteristic	Tuesday (n=972), n (%; 95% CI)	Friday (n=971), n (%; 95% CI)	Risk difference, % (95% CI)
Access	78 (8.0; 6.4 to 9.9)	70 (7.2; 5.7 to 9.0)	0.8 (–1.5 to 3.2)
Response	61 (6.3; 4.8 to 8.0)	62 (6.4; 4.9 to 8.1)	–0.1(–2.3 to 2.1)
Completion	58 (6.0; 4.6 to 7.6)	60 (6.2; 4.7 to 7.9)	–0.2 (–2.3 to 1.9)

## Discussion

### Principal Findings

This study was the first to investigate the effect of emphasizing the incentive in the subject line of the invitation email and the day of the week of sending the invitation email on physicians’ participation in a Web-based study. The cumulative response rate was 6.3% (123/1943). We found that emphasizing the incentive in the subject line of the invitation email did not significantly improve outcomes such as the response rate, completion rate, or access rate. Likewise, sending the invitation email on Tuesday or Friday did not affect the outcomes.

Previous studies reported conflicting results related to the effect of emphasizing incentives in the subject line on response rates [12-14]. In studies that found a significant effect of the subject line on the response rate of Web-based studies, the magnitude of the effect was about 1.1 to 1.2 in the risk ratio [8,13]. Thus, one possible explanation is that our study was underpowered due to the very low control response rate. There is, however, less practical meaning in the risk ratio of 1.1 in the situation of the expected response rate of 4%, such as in this study. Our sample size was large enough to detect the practically meaningful difference in the risk ratio of 1.6 to 1.7. The second possible reason is that the incentive mentioned in the subject line was not attractive enough to motivate primary physicians to respond to the invitation. Our subject line mentioned a drawing for a US $30 Amazon gift card, which might be less attractive than incentives paid in cash or by check which can be used anywhere. Furthermore, lottery incentives were reported to be less effective than fixed incentives [22]. The third possible reason is the effect of the control subject line mentioning the support from the JPCA. The authority effect might have reduced the between-group difference in outcomes with regard to the subject line.

Regarding the day of the week of sending the invitation email, our results were consistent with those of previous studies concerning postal invitation. Sending the invitation email on Tuesday evening or Friday evening did not affect the study participation of the primary care doctors in the Web-based study. Pit et al [15] synthesized the results of 3 studies and reported no significant difference in doctors’ participation in paper-based studies based on the day of the week on which the postal mails were sent or received. Our results suggest that the day of the week an invitation email is sent does not increase doctors’ participation in Web-based studies, similar to postal mail invitations for paper-based studies.

The 2 interventions about invitation emails failed to trigger primary care physicians’ response. The cumulative response rate of 6.3% is low, but it is not uncommon in Web-based studies for physicians [1]. Our results suggest that using additional media such as postal mail, telephone, or social media may be needed to attain a large enough response rate [23,24].

### Strengths and Limitations

There are several limitations to this study. One is its generalizability. All the participants were primary care physicians in Japan; therefore, it is unclear whether our findings can be applied to those in other countries. Furthermore, primary care physicians, who were the target population of this study, are known to respond to surveys less than physicians in other specialties [25]. Our results, therefore, may not be generalizable to physicians in other specialties. There were additional limitations related to the interventions. In terms of subject lines, we showed that mentioning a lottery for an Amazon gift card (US $30) as an incentive was not effective. However, we could not clarify the effect of subject lines emphasizing larger monetary incentives paid in cash or by check than ours, as $30 may not have been considered attractive enough by Japanese physicians. As for the effect of the sending day, we only compared Tuesday to Friday; therefore, the effect of other days of the week remains unclear.

However, there are also several strengths to this study. This is the first study to investigate the effect of 2 invitation email strategies targeting physicians. As participation rates in Web-based surveys for physicians are decreasing by year, our findings would provide important information when designing Web-based surveys, especially for primary care physicians. In addition, we used a rigorous RCT design, which avoided the risk of bias as much as possible. Furthermore, our sample size was large enough to detect a practically meaningful difference between the groups.

### Conclusion

Neither emphasizing a monetary incentive in the subject line of the invitation email nor varying the sending day of the invitation email increased the response rate in a Web-based physician study. Further studies are needed to find an effective invitational strategy using multimodal media beside email to improve response rates of physicians in Web-based studies.

## Appendix

Multimedia Appendix 1 CONSORT‐EHEALTH checklist (V 1.6.1).

## Abbreviations

DOCTOR: Do Overstated Conclusions Trick Our Readers?
IRB: institutional review board
JPCA: Japan Primary Care Association
RCT: randomized controlled trial
